# Supplementary material for: Deletion of wfs1 Impairs Oligodendrocyte Precursor Cells Dorsal Distribution and Myelination Through the wfs1-hmgcs1 Axis in Zebrafish
Source: Biology (Basel). 2026 Aug 16;15(16):1402. doi: 10.3390/biology15161402 (PMC13510151; doi:10.3390/biology15161402)
Supplement: Supplementary file 1 [file biology-15-01402-s001.zip › File S1-Transcriptome sequencing data and differentially expressed genes (DEGs) in wfs1-- zebrafish at 5 dpf/2.KEGG/wfs1KOvsWT/UP/src/dre00310.html]

dre00310


- dre:393860

- Up regulated genes

393860(1.20497977076277)

- dre:572649

- Up regulated genes

572649(3.62356242734646)

- dre:100003615

- Up regulated genes

100003615(1.9261094424035)
- dre:402830

- Up regulated genes

402830(1.02513538907339)
- dre:556535

- Up regulated genes

556535(2.06847796372096)

- dre:556077

- Up regulated genes

556077\_1(2.14312060824174)

- dre:100003615

- Up regulated genes

100003615(1.9261094424035)
- dre:402830

- Up regulated genes

402830(1.02513538907339)
- dre:556535

- Up regulated genes

556535(2.06847796372096)

- dre:556077

- Up regulated genes

556077\_1(2.14312060824174)

- dre:100003615

- Up regulated genes

100003615(1.9261094424035)
- dre:402830

- Up regulated genes

402830(1.02513538907339)
- dre:556535

- Up regulated genes

556535(2.06847796372096)

- dre:368239

- Up regulated genes

368239(1.14034759711527)

Close
